# Inter-Platform comparability of microarrays in acute lymphoblastic leukemia

**DOI:** 10.1186/1471-2164-5-71

**Published:** 2004-09-23

**Authors:** Stephanie A Mitchell, Kevin M Brown, Michael M Henry, Michelle Mintz, Daniel Catchpoole, Bonnie LaFleur, Dietrich A Stephan

**Affiliations:** 1 Research Center for Genetic Medicine, Children's National Medical Center, Washington, D.C. 20010, USA; 2 Institute of Biomedical Sciences, George Washington University Medical Center, Washington, D.C. 20037, USA; 3 Family Studies, Translational Genomics Research Institute, Phoenix, AZ 85004, USA; 4 Department of Hematology and Oncology, Children's National Medical Center, Washington, D.C. 20010, USA; 5 The Children's Hospital at Westmead, Westmead, Australia; 6 Department of Preventative Medicine, Vanderbilt University Medical Center, Nashville, Tennessee 37232, USA; 7 Neurogenomics Program, Translational Genomics Research Institute, Phoenix, AZ 85004, USA

## Abstract

**Background:**

Acute lymphoblastic leukemia (ALL) is the most common pediatric malignancy and has been the poster-child for improved therapeutics in cancer, with life time disease-free survival (LTDFS) rates improving from <10% in 1970 to >80% today. There are numerous known genetic prognostic variables in ALL, which include T cell ALL, the hyperdiploid karyotype and the translocations: t(12;21)[*TEL-AML1*], t(4;11)[*MLL-AF4*], t(9;22)[*BCR-ABL*], and t(1;19)[*E2A-PBX*]. ALL has been studied at the molecular level through expression profiling resulting in un-validated expression correlates of these prognostic indices. To date, the great wealth of expression data, which has been generated in disparate institutions, representing an extremely large cohort of samples has not been combined to validate any of these analyses. The majority of this data has been generated on the Affymetrix platform, potentially making data integration and validation on independent sample sets a possibility. Unfortunately, because the array platform has been evolving over the past several years the arrays themselves have different probe sets, making direct comparisons difficult.

To test the comparability between different array platforms, we have accumulated all Affymetrix ALL array data that is available in the public domain, as well as two sets of cDNA array data. In addition, we have supplemented this data pool by profiling additional diagnostic pediatric ALL samples in our lab. Lists of genes that are differentially expressed in the six major subclasses of ALL have previously been reported in the literature as possible predictors of the subclass.

**Results:**

We validated the predictability of these gene lists on all of the independent datasets accumulated from various labs and generated on various array platforms, by blindly distinguishing the prognostic genetic variables of ALL. Cross-generation array validation was used successfully with high sensitivity and high specificity of gene predictors for prognostic variables. We have also been able to validate the gene predictors with high accuracy using an independent dataset generated on cDNA arrays.

**Conclusion:**

Interarray comparisons such as this one will further enhance the ability to integrate data from several generations of microarray experiments and will help to break down barriers to the assimilation of existing datasets into a comprehensive data pool.

## Background

The advent of DNA microarrays has provided the science community with a tool to concurrently examine the expression of thousands of genes within a given cell or tissue type, thus providing a platform for future diagnoses and prognostic analyses of disease with gene-level specificity [1,2]. Microarray technology is progressing rapidly as better sequencing and prediction algorithms allows for more refined gene prediction. This has prompted the evolution of the probe sets contained within the array chips over the past few years, in both oligonucleotide and cDNA arrays [3]. This microarray platform expansion has hindered the direct comparison between numerous datasets of a given phenotype that have been produced using several generations of arrays. In microarray analyses of disease, having a large number of samples better accounts for the biological variability between individuals and therefore increases the power to enhance and define a pathogenetic model for that disease. Due to the considerable expense of microarray chips and the equipment required, along with the common problem of sufficient sample acquisition, being able to combine and compare datasets from various laboratories and across all microarray generations would be a benefit to the entire biomedical community. The constant evolution of microarrays has thus resulted in a significant hindrance to their power as a research or diagnostic tool by dividing datasets according to platform and seemingly limiting their interarray comparability.

With the large number of microarray datasets available in the public domain for distinct disease phenotypes from various microarray platforms, cross-platform comparisons can currently be attempted. For example, a number of laboratories have been studying diagnostic pediatric acute lymphoblastic leukemia (ALL) samples from human bone marrow on both oligonucleotide and cDNA microarrays and depositing the raw intensity values into the public domain. Additionally, in 2002 at St. Jude Children's Research Hospital, Yeoh *et al*. generated a list of genes that have distinct expression levels for various karyotypic and phenotypic aberrations common to pediatric ALL [4]. This set of genes has been useful as a prognostic profile for ALL by identifying subclasses of the cancer using microarray technology [4]. Importantly, the St. Jude's gene list has been validated on independent datasets both within their own lab and in an independent laboratory by Kohlmann *et al*. in 2004. They used the Yeoh *et al *gene list to successfully segregate the various subclasses of adult ALL [5]. Consequently, this disease provides an excellent model for testing interarray comparability using one common gene list.

ALL is the most common pediatric malignancy comprising over 75% of the annual diagnoses of leukemias in children [6]. In the United States, the outcome for children with ALL has improved dramatically over the past thirty years with the long-term disease-free survival (LTDFS) rates increasing from less than 10% in 1970 to over 80% today [7]. However, ALL still carries the risk of relapse in over 20% of patients [8]. ALL survival is largely due to a greater understanding of the risk factors that affect outcome, which has allowed for more intensity-tailored treatment following an assessment of the patient's risk [7]. Accurate segregation of patients into their proper risk group is critical to allow for a risk-stratified treatment that is effective enough to clear the disease and decrease the risk of relapse while minimizing the negative long-term side effects [7]. Factors that affect prognosis are age, sex, race, white blood cell count at diagnosis, phenotypic differences, such as T-cell versus B-cell lineage ALL and karyotypic alterations, such as the hyperdiploid karyotype and the translocations t(12;21)[*TEL-AML1*], t(4;11)[*MLL-AF4*], t(9;22)[*BCR-ABL*], and t(1;19)[*E2A-PBX*] [7,9]. These genetic lesions can affect the individual's response to treatment. For example, patients with a hyperdiploid karyotype and those with the *TEL-AML1 *fusion gene have a better prognosis than patients in the other subclasses [10]. The initial diagnosis and classification of ALL is currently revealed through multiple time-consuming and expensive tests often involving multiple laboratories [11]. Thus, a tool that could consolidate these tests into one diagnostic platform would be beneficial to both researchers and clinicians working with ALL.

In this study, we sought to determine if datasets from different microarray platforms could be compared in a useful manner. We chose to study pediatric ALL because there is already a substantial pool of datasets freely available in the public domain. First, we collected pediatric ALL array data and cDNA array data generated in experiments from various laboratories. In addition to the collection of public data, we supplemented the hyperdiploid karyotype and the T-cell lineage ALL subclasses by expression profiling additional diagnostic pediatric ALL samples from a tumor bank in Children's National Medical Center in Washington, D.C. We used these independent datasets, including the one generated in our lab, to validate the gene predictors, as defined by Yeoh *et al*. (2002), for each of the aforementioned prognostic genetic variables. Cross-platform array validation was used successfully to ascertain the accuracy, sensitivity and specificity of the gene predictors for the prognostic variables. In addition, we have demonstrated the ability to compare datasets from different microarray platforms. To our knowledge, this is among the first known successful applications of this technique, along with the validation of the Yeoh *et al *pediatric ALL gene lists on adult ALL by Kohlmann, *et al *(2004) [5]. Interarray comparisons such as these will further enhance the ability to integrate data from several generations of microarray experiments and will help to break down barriers to the assimilation of existing datasets into a comprehensive data pool.

## Results and discussion

### Expression profiling of ALL diagnostic bone marrow

To supplement the ALL subclasses that are under-represented in expression profiling thus far, we collected and extracted the total RNA from sixteen diagnostic bone marrow samples housed at Children's National Medical Center; seven of the hyperdiploid karyotype and nine with T-cell lineage. The extracts were then hybridized to Affymetrix U133A arrays and expression profiled as an independent training data set.

### Validation of the gene predictors using independent datasets spanning various array platforms

In order to validate the portability of gene predictors across microarray platforms we compared the accuracy with which the six prevalent ALL subclasses can be distinguished on disparate array platforms. To do this we used the discriminating gene lists (~40 genes), which were provided by the comprehensive training ALL sample set analyzed and published by Yeoh *et al*. in 2002 [4]. In their study they hybridized RNA from ALL bone marrow samples to Affymetrix U95Av2 arrays. The resulting expression data were analyzed by multiple statistical methods to facilitate the generation of lists of genes that represent the greatest difference in expression between the ALL subclasses [4]. Yeoh *et al*. used both a training and test dataset in their analysis to first uncover the subclass-specific gene expression profiles and then to test their predictability on independent samples [4]. The genes are listed hierarchically, along with supplemental information about the statistical methods used, at . We then accumulated the ALL diagnostic bone marrow array data available in the public domain (Table 1).

**Table 1:** Training and test datasets used to validate ALL subclass predictors

**Training Sample Set**	**Microarray Platform **	
Yeoh *et al*. *Cancer Cell*, 2002, **1:**133–143	Affymetrix HG_U95Av2	

**Validation Sample Set**	**Microarray Platform**	**Predictors**

Armstrong *et al*. *Nat. Genet*., 2002, **30(1):**41–7	Affymetrix HG_U95Av2	Hyperdiploid, *MLL-AF4*, *TEL-AML1*
Mitchell *et al*. Unpublished data 2003	Affymetrix HG_U133A	Hyperdiploid, T-ALL
Stephan DA, Golub TR. Unpublished data 2000	Affymetrix HuGene FL	*TEL-AML1, E2A-PBX1*
Golub *et al*. *Science*, 1999, **286:**531–7	Affymetrix HuGene FL	T-ALL
Ramaswamy *et al*. *Proc. Natl. Acad. Sci. USA*, 1999, **98(26):**15149–54	Affymetrix Hu6800 and Hu35KsubA	T-ALL
Moos *et al*. *Clin. Cancer Res*., 2002, **8:**3118–3130	cDNA	*TEL-AML1, MLL-AF4, BCR-ABL1*, T-ALL
Catchpoole *et al*. Unpublished data 2002.	cDNA	T-ALL

The independent datasets that we accumulated, including the one generated in our lab, spanned four different microarray platforms: Affymetrix HuGene FL, U95Av2, U133A and custom cDNA microarray platforms (Table 1). To modify these test datasets into data that could be directly applied to the predictor gene lists from the U95Av2 arrays, we correlated the probe numbers between these different arrays and the U95Av2 set using the probe match spreadsheet, NetAffx, available at . We then used the discriminating gene list for each subclass to extract the appropriate probes and their intensity values from the expansive expression data for each sample of the validation datasets independently. The level of similarity between the probe sets of the two different array platforms was evident through the number of genes within the 40 discriminators that could be found within the validation data (Table 2). For example, the data published by Armstrong *et al*. (2002) was generated on the U95Av2 array platform [6]. Therefore, expression data for all 40 predictor genes could be correlated and represented in their corresponding *MLL-AF4*, *TEL-AML1*, and hyperdiploid datasets. Similarly, the U133A arrays that were used to generate expression data in our lab for the hyperdiploid karyotype and the T-cell lineage ALL contained probes representing the majority of the 40 discriminators, with 38 and 35 genes, respectively. The HuGene FL arrays contain significantly fewer probe sets in common with the selected predictors (from the later-generation U95Av2 microarrays). Accordingly, of the 40 original predictor probes, only 25 were present in the *TEL-AML1 *dataset, 26 in the *E2A-PBX1 *dataset and 13 in the T-cell dataset. The difficult task of matching probes from the Affymetrix gene chips with cDNA arrays was illustrative of the disparities between the probe sets within these two platforms. For example, the five cDNA predictors built using the datasets produced by Moos *et al*. (2002) and Catchpoole *et al*. (unpublished data) contained data for only ten genes or less from the predictor set gene list.

**Table 2:** Prediction accuracies for ALL subclasses as determined by the different microarray platforms.

**ALL Subclass**	**Microarray Platform**	**# of Samples in the Dataset**	**# of Samples Representing the Predictor Subclass**	**# of Genes in Predictor (out of 40)^1^**	**Accuracy (%)^2^**	**Sensitivity (%)^3^**	**Specificity (%)^4^**
**Hyperdiploid**	Affymetrix U95Av2	43^a^	5	40	97	80	100
**Hyperdiploid**	Affymetrix U133A	16^b^	7	38	94	86	100
**T-ALL**	Affymetrix U133A	16^b^	9	35	100	100	100
**T-ALL**	Affymetrix HuGene FL	41^c^	8	13	100	100	100
**T-ALL**	Affymetrix Hu6800	20^d^	10	30	95	100	90
**T-ALL**	cDNA	52^e^	7	5	98	86	100
**T-ALL**	cDNA	9^f^	3	29	100	100	100
**TEL-AML1**	Affymetrix U95Av2	43^a^	9	40	91	67	97
**TEL-AML1**	Affymetrix HuGene FL	23^g^	14	30	86	79	100
***TEL-AML1***	cDNA	52^e^	12	10	87	83	88
**MLL-AF4**	Affymetrix U95Av2	43^a^	20	40	100	100	100
***MLL-AF4***	cDNA	52^e^	2	7	98	50	100
***E2A-PBX1***	Affymetrix HuGene FL	23^c^	2	26	96	50	100

^1 ^With a few exceptions, the majority of the gene lists published by Yeoh *et al *(2002) contain 40 genes.

^2 ^The ability of the predictor to correctly classify the blinded test set into the correct subgroup

^3 ^(# of positive samples predicted correctly)/(total #of true positives)

^4 ^(# of negative samples predicted correctly)/(total #of true negatives)

^a ^Armstrong et al. (2002) *Nat. Genet*. **30(1)**, 41–7.

^b ^Mitchell et al. (2003) Unpublished data.

^c ^Golub et al. (1999) *Science ***286**, 531–7.

^d ^Ramaswamy et al. (2001) *PNAS ***98(26)**, 15149–54.

^e ^Moos et al. (2002) *Clin Cancer Res*. **8**, 3118–3130.

^f ^Catchpoole et al. Unpublished data.

^g ^Stephan et al. (2000) Unpublished data.

**Figure 1:**
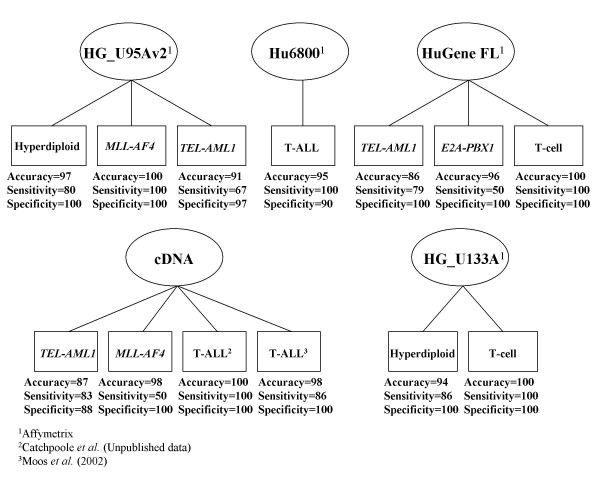
Summary of results of the various ALL subclass predictors tested. The predictors are organized according to microarray platform and the results are listed under each class in terms of the accuracy, sensitivity and specificity of the classification.

To validate the gene predictors from Yeoh *et al*. (2002), using the aforementioned independent test datasets from various array platforms, we employed supervised learning methods using GeneCluster2 software. Prior to analysis, we formatted the discriminating gene expression values from the test datasets onto spreadsheets according to software instruction, and subsequently applied the data to the software. Genecluster2 then generated blinded predictions on the ALL samples of the test datasets through weighted voting with a leave-one-out methodology. This is accomplished by randomly removing one sample at a time from the test dataset of ALL samples and "training" a predictor gene profile to recognize similarities or disparities between the two classes based on the expression profiles of the samples for the genes of interest [11]. In this manner each sample is assigned to one of the two classes based on their expression pattern of the predictor genes. The prediction accuracy, sensitivity and specificity were calculated for each of the predictors from each array platform and are displayed in both figure 1 and table 2. The accuracy of our predictors ranged from 86%–100%, with a mean accuracy of 95%. The mean specificity of the predictors was 98%, ten of which provided a specificity of 100%. The sensitivity ranged from 50%–100%. The mean sensitivity was 83% (fig. 1, table 2).

We saw a high accuracy from the predictors employing data from both U95Av2 and U133A arrays, attesting to the fact that nearly all of the 40 discriminating genes were present in the datasets, thus maximizing the possible prediction strength. In the case of the *E2A-PBX1 *predictor (96%) and the *MLL-AF4 *predictor (98%), the sensitivities were only 50%. In both cases there were only 2 samples out of the sample pool expressing the respective translocation and in both analyses one of the two was continuously classified incorrectly. This could be due to many factors, misdiagnosis or mislabelling of the sample, poor sample quality or differences in sample handling. It is difficult to draw a conclusion due to the fact that the samples were collected and processed in a laboratory outside of our own. Another problem with these two predictors may simply be the low sample number. Two samples may not provide enough strength to the classification by Genecluster2 simply due to the inability of such a low sample number to account for the biological variability that exists between patients that is independent of their subclass of ALL. The most surprising result was the high accuracy with which the gene lists could classify T-ALL, *TEL-AML1 *and *MLL-AF4 *from cDNA data considering the disparity between the probe sets of cDNA arrays and oligo arrays. The accuracies of the classifiers were: T-ALL (Catchpoole data), 100%; T-ALL (Moos data), 98%; *MLL-AF4*, 98%; and *TEL-AML1*, 87%. Therefore, it appears that the number of genes in the predictor gene list is much less of a factor in the predictor's classification accuracy than the number of samples representing the phenotype of interest, which supports the argument that being able to do cross-platform analyses to increase sample size is crucial for sensitive and specific class prediction using expression data. This is strongly illustrated by the cDNA predictors, which have few probes in common with the arrays used to generate the predictor gene list, but still classify the ALL samples with high sensitivity, specificity and accuracy. On the other hand the *E2A-PBX1 *predictor and the *MLL-AF4 *predictor had low sensitivities correlating to a low sample number in these groups. The high number of probes in common between the arrays used to generate these independent datasets and the arrays used to generate the predictor gene list, 26/40 and 40/40, respectively, were not able to rescue the low sensitivity of the predictor.

## Conclusions

Currently the vast majority of expression data from numerous labs is not being used to its highest potential as independent labs continue to move to more expansive array platforms rendering older datasets less informative in the context of new data. Increasingly, progressive technologies in genome databasing and chip construction are prompting this inevitable evolution of microarrays. Until data can be analyzed and directly compared across array platforms, the size of the data pools will remain small and isolated according to platform [12]. Here we have shown that the previously validated predictor gene list from Yeoh *et al*. (2002) withstands validation by testing the predictors using a leave-one-out strategy on all publicly available datasets as well as a dataset generated in our own lab regardless of the array platform used. This meta-data analysis of over 200 arrays from diagnostic ALL samples with hyperdiploidy, T-cell lineage and translocation status (previously confirmed through gold standard techniques), shows that expression profiling as an integrated platform is robust and that ALL data, and presumably other disease models, can be interplatform comparable. By validating the comparability between data from distinct microarray platforms we have demonstrated a tool that can enhance the statistical power provided by large sample sets. Thus, we can potentially develop and validate sensitive diagnostic tools based on large training sample sets, to allow for the rapid assignment of individualized therapy to improve disease outcome in pediatric ALL and other diseases.

## Methods

### RNA extraction from bone marrow samples

ALL diagnostic bone marrow samples were housed in a tumor bank in Children's National Medical Center in Washington, D.C. Mononuclear cells from diagnostic bone marrow aspirates were separated using density centrifugation on Cappel Lymphocyte Separation Medium (ICN Biomedicals, Aurora, Ohio) and immediately flash frozen according to manufacturer's instruction. A total of sixteen samples were obtained with IRB approval; seven with a hyperdiploid karyotype and nine samples of a T-cell lineage as confirmed by immunophenotyping. The frozen samples were placed directly in TRIzol reagent for RNAse-free thawing for total RNA extraction. We extracted a 10 μg–20 μg pellet of total RNA from each sample by centrifugation following phenol-chloroform extraction. The integrity of the resultant total RNA from each sample was quantified by gel electrophoresis before it was considered to be of good quality for cDNA synthesis. Samples were re-extracted if ribosomal bands were not visible.

### Expression profiling and support vector machine meta-analysis

10 μg of the extracted RNA from each sample was labelled and hybridized to an Affymetrix U133A array (Affymetrix, Santa Clara, CA) according to protocol as previously described [13]. Intensity values were calculated using Microarray Suite 5.0 (MAS 5.0) and expression values were adjusted to fall within the lower and upper limits of 1 and 45000 as described by Yeoh *et al*. (2002) [4]. To create a predictor that allows for the direct comparison between different generation Affymetrix arrays and cDNA arrays, we used the predictor gene list for each subclass provided by Yeoh *et al*. (2002) from Affymetrix U95Av2 microarrays. The 40 genes that showed the greatest mean difference in expression between the subclass of interest and the remaining subclasses was used as our predictor gene set. The gene lists and additional information, including the statistical metrics used to generate the gene list from the training set, can be viewed at: . To identify comparable data points between the gene lists from the training set (produced on the U95Av2 Affymetrix chip), and the expression values of samples provided by other public datasets on different generation Affymetrix arrays, we used the probe match function within NetAffx . Data for these probe pairs in the validation sets were extracted and expression values were linearly adjusted to fall within 1–45000 [4]. Affymetrix probes were identified within cDNA data by a combination of BLAST sequence comparison  and GenBank accession number queries. Ratios were log-transformed prior to analysis. GeneCluster2 (; Center for Genome Research, MIT, Cambridge, MA) was used to perform blinded predictions on the validation dataset using weighted voting with a leave-one-out methodology. Accuracy, specificity and sensitivity values were then generated for each predictor, as a measure of the predictor's ability to correctly group the samples into their respective class in the validation sets.

## Authors' contributions

SAM carried out the accumulation of datasets, preparation of the predictors, data analysis and drafted the manuscript. SAM with the help of KMB and MMH participated in the sample preparation and hybridization to arrays of the in-house gene expression data. KMB also trained SAM and participated in the preparation of the predictors with SAM and also provided essential mentor support. MM participated in finding and extracting the public data and aided in drafting the manuscript. MMH was a tremendous help in editing the final manuscript. BL provided statistical guidance throughout the project. DC provided us with cDNA array data and aided in the analysis of the cDNA expression data. DC also provided much appreciated critical input throughout the entire project. DAS initiated and crafted the idea and provided the necessary mentorship along the way.
